# Clinical courses of 24,563 hospitalized COVID-19 patients during the first 12 months of the pandemic in the Central City of Iran

**DOI:** 10.1038/s41598-023-32292-2

**Published:** 2023-04-21

**Authors:** Seyedeh Mahideh Namayandeh, HamidReza Dehghan, Mohammad Hassan Lotfi, Mohammad Reza Khajehaminian, Saeed Hosseini, Vali Bahrevar, AliAkbar Jarrahi, Fatemeh Majidpour

**Affiliations:** 1 Clinical Research Development Center, Afshar Hospital, Shahid Sadoughi University of Medical Sciences, Yazd, Iran; 2 Research Center for Health Technology Assessment and Medical Informatics, School of Public Health, Shahid Sadoughi University of Medical Sciences, Yazd, Iran; 3 Center for Healthcare Data Modeling, Department of Biostatistics and Epidemiology, School of Public Health, Shahid Sadoughi University of Medical Sciences, Yazd, Iran; 4 Department of Health in Emergency and Disaster, School of Public Health, Shahid Sadoughi University of Medical Sciences, Yazd, Iran; 5 Department of Health Education & Health Promotion, Shahid Sadoughi University of Medical Sciences, Yazd, Iran; 6 Deputy for Treatment, Shahid Sadoughi University of Medical Sciences, Yazd, Iran

**Keywords:** Medical research, Epidemiology

## Abstract

This study was designed and implemented to analyze and establish documents related to the above cases in the first to third COVID-19 epidemic waves for the use of researchers and doctors during and after the epidemic. The current case series study was conducted on 24,563 thousand hospitalized COVID-19 patients by examining their clinical characteristics within a one-year period from the beginning of the pandemic on 02.22.2020 to 02.14.2021, which included the first to the third waves, based on gender and severity of COVID-19. The mean age of the participants was 56 ± 20.71, and 51.8% were male. Out of a total of 24,563 thousand hospitalized COVID-19 patients until February 2021, there were 2185 mortalities (9.8%) and 2559 cases of severe COVID-19 (13.1%). The median length of hospitalization from the time of admission to discharge or death in the hospital (IQR: 13–41) was estimated to be 21 days. The rate of hospital mortality was higher in severe (37.8%) than in non-severe (4.8%) cases of COVID-19, While the risk of severe cases increased significantly in the third (HR = 1.65, 95% CI: 1.46–1.87, *P* < 0.001) and early fourth waves (HR = 2.145, 95% CI: 1.7–2.71, *P* < 0.001). Also, the risk of contracting severe COVID-19 increased significantly in patients aged ≥ 65 years old (HR = 2.1, 95% CI 1.1.93–2.72, *P* < 0.001). As shown by the results, the rates of hospital mortality (9.3% vs. 8.5%) and severe cases of COVID-19 (13.6% vs. 12.5%) were higher among men than women (*P* < 0.01). In our study, the mortality rate and severity of COVID-19 were within the scope of global studies. Men experienced higher severity and mortality than women. The was a significantly higher prevalence of old age and underlying diseases in individuals with severe COVID-19. Our data also showed that patients with a previous history of COVID-19 had a more severe experience of COVID-19, while most of these patients were also significantly older and had an underlying disease.

## Introduction

Two years after the start of the COVID-19 ^1^ pandemic in December 2019 in China^2^ and despite the injection of 12,355,390,461 doses of COVID-19 vaccine until August 12, 2022 (https://covid19.who.int)^3^, adherence to preventive behaviors^4^, increased transmission of new strains of COVID-19 virus^4^, and relocation of outbreaks^5^ have led to an increase in the prevalence of COVID-19 worldwide^3^. The first COVID-19 case in Iran was detected on 19 February 2020 in Qom^6^. Since the start of the covid 19 pandemic in iran to 15 December 2022, 7,560,162, confirmed cases of COVID-19, including 144,658 deaths have happened in Iran(Referto https://covid19.who.int/). A study reported the prevalence of confirmed COVID-19 cases,hospital case fatality rate and mortality were reported 0.019, 156 and about 8.2 per 100,000 respectively in Yazd province^7^.

Regarding disease severity, 80% of cases are asymptomatic and 20% are severe and critical^8^. In a meta-analysis study in 2021, the prevalence of severe and critical cases was 17.84% and 4.9%, respectively^9^. According to the results of meta-analysis studies (2021), the most common clinical symptoms in patients with COVID-19 are fever (83%), cough (60%), and dyspnea (42%)^10,11^, while the most common underlying diseases of hypertension, obesity, diabetes, and cardiovascular disease have also been reported^10,12^. About 50% of hospitalized patients, 70% of patients admitted to the intensive care unit^13^, and 84.1% of the fatal cases of COVID-19 had at least one or more underlying diseases^14^. With the change of COVID-19 strains, the prevalence of symptoms, transmissibility^15^, and the complications and severity of mortality of this disease has changed^12^. Also, the prevalence of underlying diseases, incidence, severity, and mortality of COVID-19 vary from region to region^12^, and it is not clear which clinical signs provide more information in the COVID-19 diagnosis^16^. However, such a knowledge can lead to a better understanding of the factors affecting the severity and mortality and epidemiological changes of the infection^17^ and help to improve care in high-risk groups^18^. To know more about the disease and ensure the implementation of new health interventions to control the prevalence of new strains of this disease^17^, we need to highlight the clinical features of patients with COVID-19 based on the data of the infected cases. This case series study was conducted on 24,563 COVID-19 hospitalization patients for the first time in this area. It evaluated in-hospital outcomes in natural settings during the first year of the COVID-19 pandemic in a real crisis. With increasing referrals to hospitals and lack of PCR kits, low sensitivity and specificity of rapid tests, a large number of patients waited for admission. So more physicians had been forced to admit more patients only based on their clinical judgment. Finally, this article investigated, besides clinical and paraclinical characteristics of COVID- 19 patients, tried to evaluate in-hospital outcomes according to admission indications criteria in real crisis; on the other hand, the first COVID-19 pandemic, we assess the natural history of admitted COVID-19 patients because of COVID-19 vaccine any special treatment hadn't been established yet.

## Materials and methods

**Figure 1:**
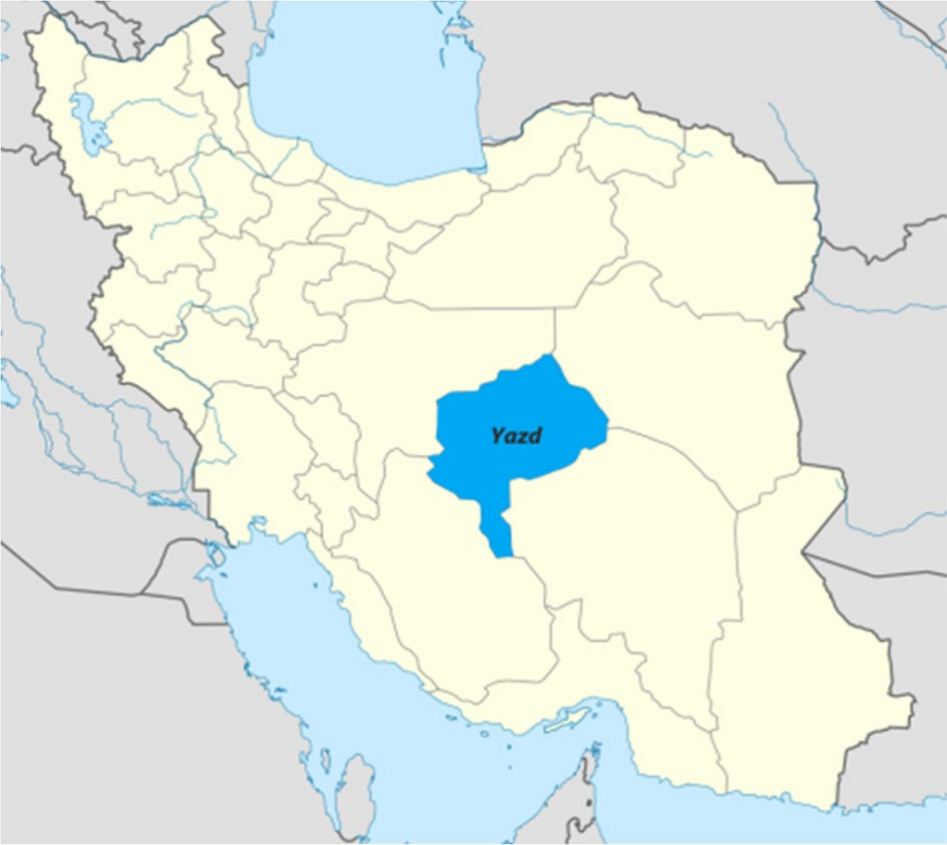
Map of Iran with location of Yazd Area (Yazd province is shown in Highlight, From Wikipedia, the free encyclopedia, https://en.wikipedia.org/wiki/File:Iran_location_map.svg).

Yazd province is located in the central part of Iran (Fig. 1), The historic city of Yazd was inscribed on Unesco's World Heritage List in July, 2017. Yazd is known as the oldest earthen city in the world and the second most historic city of the world after Venice in Italy. We uploaded iran's map From Wikipedia and we used Paint 3D software and marked Yazd province in it.

### Study design and patients

The present case-series study aimed to investigate the demographic-clinical characteristics of 24,563 patients admitted to the COVID-19 hospitals in Yazd province based on gender and severity of the disease for approximately one year from February 22, 2020 (the beginning of the pandemic in Iran) to February 13, 2021.

### Data collection and measurement of variables

The current study used medical records and information of the patients with COVID-19 recorded in the Medical Care Monitoring Center (MCMC) system of Yazd province, including demographic data, history of exposure to people with COVID-19, recurrent history of COVID-19 symptoms, underlying conditions, patient status at admission, treatments and physical examinations (including pulse oximetry), chest CT scan, and hospital outcomes.

Hospitalized patients were classified as severe and non-severe. Severe cases of COVID-19 were considered based on one of the following criteria: ≤ 85% blood oxygen level or airway intubation.

### Statistical analysis

Demographic-clinical characteristics of patients admitted to the hospital due to COVID-19 were reported using descriptive statistics (mean ± SD), median, and frequency (%). Demographic-clinical characteristics of hospitalized patients were described and compared by sex and severity of the COVID-19 disease using chi-square, t-test, and ANOVA or its non-parametric equivalent. We used of log-rank test to comparison different median hospital survival in patients and performed cox model for to assess the association between variables with dependent variable. The primary event of interest was in-hospital mortality, severity. The significance level of the test was considered to be 5%. Analysis was performed using R software version 4.2.2 and IBM SPSS Statistics for Windows, Version 26.0. software.

### Patient and public involvement

Patients and/or the public were not involved in design, plan, manage and carry out research.

### Ethics approval and consent to participate

The present study was ethically approved by the ShahidSadoughi University of Medical Sciences’ ethics committee (ethics code: IR.RUMS.REC.1399.017). All provisions of the Declaration of Helsinki adhered in our study, especially appropriate ethical and scientific review. All patients signed a predefined consent specially their clinical data usage.

## Results

Overall, 24,563 hospitalized patients with COVID-19 were admitted within 12 months. The mean age of these patients was 56. 20 ± 20.71 years and in the range of (0–127) years (infant less than one year). Sexual distribution was male (51.5%), and 95.3% were Iranian. Out of the population under study, 4.85% were referred to the hospital in person, and 9.9% were transferred to the hospital using the services of the emergency medical system.

Demographic and clinical characteristics of patients by gender are presented in Table 1.

**Table 1:** Clinico-demographic profile and hospital outcomes of 24,563 admitted COVID-19 patients during 1 year based on gender.

	Total	Male	Female	*P*-value
Age				
< 65 yr	15,661(63.8)	8366(34.2)	7295(38.3)	
≥ 65 yr	8879(36.2)	4354(65.8)	4525(61.73)	< 0.001*
COVID-19 History(yes)	165(1.3)	78(1.2)	87(1.4)	0.19
Exposure to the COVID-19 patient (yes)	8533(34.7)	4348(34.2)	4185(35.4)	0.045*
The onset of the admission time(days)	4.89 ± 6.38	4.96 ± 7.15	4.82 ± 5.41	0.07
Breath rate				
14–18	5249(46.9)	2735(46.9)	2514(46.9)	
18–22	4753(42.5)	244(49.1)	2309(43.1)	
22–28	996(8.9)	544(9.3)	542(8.4)	
≥ 28	189(1.7)	104(1.8)	85(1.6)	0.25
Mean temperature-°C	37.11 ± 0.74	37.12 ± 0.76	37.09 ± 0.72	0.01*
Symptoms number (%)				
Fever	10,054(40.9)	5341(42)	4713(39.8)	0.001*
Cough	11,046(45)	5381(44.5)	5381(45.5)	0.12
Dyspnea	11,009(44.8)	5763(45.3)	5246(44.3)	0.14
Chest pain	1078(4.8)	518(4.4)	560(5.2)	0.007*
Abdominal pain	401(50.8)	43(3.3)	34(3.7)	0.10*
Nausea	1541(6.7)	759(6.4)	782(7.1)	0.023
Vomiting	1203(5.3)	588(4.9)	615(5.6)	0.024*
Diarrhea	818(3.6)	429(3.6)	389(3.5)	0.8
Loss of appetite	2284(10)	1216(10.2)	1068(9.7)	0.22
Muscular. Pain	6941(28.3)	3467(27.2)	3474(29.4)	< 0.001*
Paresis	152(0.7)	89(0.8)	63(0.6)	0.17
Plegia	79(0.4)	49(0.4)	30(0.3)	0.07
Vertigo	614(48.5)	298(2.5)	316(2.9)	0.07
Seizure	77(0.3)	43(0.3)	34(0.3)	0.48
Insomnia	380(1.5)	189(1.5)	191(1.6)	0.41
Unconsciousness	1170(4.8)	642(5)	528(4.5)	0.03*
Headache	1824(8.1)	913(7.8)	911(8.4)	0.07
Smell impairment	380(1.5)	189(1.5)	191(1.6)	0.41
Taste impairment	225(0.9)	20(0.9)	105(0.9)	0.65
Skin sign	30(0.1)	1(0.1)	19(0.2)	0.09
GI symptom	4086(17.9)	2102(17.1)	1984(18.8)	0.4
Respiratory symptom	17,737(74.5)	9221(74.6)	8516(74.4)	0.78
Nervous system	223(0.9)	119(0.9)	104(0.9)	0.6
Comorbidity				
Cardiovascular disease	2012(8.2)	976(7.7)	1036(8.8)	0.002*
Hypertension	283(11.5)	1157(9.1)	1674(14.1)	< 0.001*
Diabetes mellitus	2853(11.6)	1253(9.8)	1600(13.5)	< 0.001*
Cancer	330(1.3)	166(1.3)	164(1.4)	0.5
COPD (Chronic Obstructive Pulmonary Disease)	504(2.1)	293(2.3)	211(1.8)	0.004*
CKD (chronic kidney disease)	393(1.6)	219(1.7)	174(1.5)	0.11
Asthma (N = 239)	239(1)	101(0.8)	38(1.2)	0.003*
Liver disease (N = 49)	49(0.2)	30(61.2)	19(38.8)	0.18
Neurological disorders	223(0.9)	119(53.4)	10,446.6)	0.64
Hematology (N = 79)	74(0.3)	33(0.3)	41(0.3)	0.2
HIV (N = 13)	13(0.1)	7(0.1)	6(0.1)	0.8
Immunodeficiency disorders				
Congenital disorder (N = 23)	23(0.1)	4(0)	19(0.2)	0.001*
Smoking	456(1.9)	434(3.4)	22(0.2)	< 0.001*
Opium	456(1.9)	404(3.2)	52(0.4)	< 0.001*
PaO_2_ saturation %				
93% >	12,243(49.8)	6361(50)	5882(49.7)	
93% ≤	12,320(50.2)	6370(50)	5950(50.3)	0.69
Early diagnosis based on				
Positive PCR	9510(38.7)	4824(37.9)	4686(39.6)	
Abnormal lung CT	8016(32.7)	4271(33.6)	3745(31.7)	
Clinically admission	7023(28.6)	3629(28.5)	3394(28.7)	0.003*
Inpatients Treatments				
Mechanical ventilation	1213(4.9)	665(5.2)	548(4.6)	0.03*
Dialysis (N = 224/393)	224(57)	113(51.6)	111(63.8)	0.015
Oxygen therapy	3949(31.2)	2066(31.2)	1883(31.2)	0.9
Chemotherapy (N = 48)	48(38.7)	20(34.5)	28(42.4)	0.3
Severity				
Yes	2559(13.1)	1398(13.6)	1161(12.5)	
No	17,017(86.9)	8893(86.4)	8124(87.5)	0.025*
Hospital Outcome				
Death	2185(8.9)	1184(9.3)	1001(8.5)	
Recovery	22,378(91.1)	11,547(90.7)	10,831(91.5)	0.02*
Hospitalization length(days)	4.89 ± 6.38	4.96 ± 7.15	4.82 ± 5.41	0.07

*P < 0.05.

Table 1 shows that most patients were young and < 65 years old, with women admitted more than men in this age group. However, men were admitted more than women in the age group $\ge$ 65 years (*P* < 0.001). About 1.3% (n = 165) of patients reported a previous history of the COVID-19 infection, with women reporting a prior history of the COVID-19 infection more than men. Yet, the recurrence of the COVID-19 infection was not affected by gender. Most patients presented with clinical complaints of abdominal pain, dizziness, cough, dyspnea, fever, and muscle aches. Women reported more chest pain, nausea, vomiting, and muscle aches, while men reported fever and unconsciousness (*P* < 0.05). The highest prevalence of underlying diseases was reported in patients with diabetes, hypertension, and cardiovascular diseases. Underlying cardiovascular disease, hypertension, diabetes, asthma, and congenital disorders were more common in women, while COPD was more prevalent in men (*P* < 0.01). Men reported a more significant history of smoking and drug use than women. According to the hospitalization indication, most patients were admitted with a positive PCR diagnostic test, the majority of whom were hospitalized (*P* < 001.0). Overall, 28.6% (7023 patients) were admitted to the hospital with a clinical diagnosis, and men were hospitalized significantly more than women in all hospitalization indications (*P* = 0.003). The mean body temperature of men was considerably higher than that of women (*P* = 0.01). Men were significantly more likely than women to be treated with ventilators and dialysis (*P* = 03.0). Severe cases of COVID-19 and mortality were significantly higher in men than women.

Demographic and clinical characteristics of patients based on the severity of COVID-19 are presented in Table 2.

**Table 2:** Clinico-demographic profile of patients based on the severity of COVID-19.

Variables	Total	Non sever	Sever	*P*-value
Age				
< 65 yr	13,139(67.2)	12,064(70.9)	1075(42.1)	
≤ 65 yr	6422(32.8)	4941(29.1)	1481(57.9)	*< 0.001
Sex				
Male	10,291(52.6)	8893(52.3)	1398(54.6)	
Female	9285(47.4)	8124(47.7)	1161(45.4)	*0.025
Covid-19 History(yes)	7514(38.4)	6407(1.1)	1107(2.5)	*< 0.001
Exposure with a covid-19 patient (yes)	165(1.3)	115(37.7)	50(43.3)	*< 0.001
Hospitalization length(days)	4.87 ± 6.64	4.5 ± 6.09	7.33 ± 9.14	*< 0.001
Breath rate				
14–18	5249(46.9)	4576(48.2)	673(39.7)	
18–22	4753(42.5)	4046(42.6)	707(41.7)	
22–28	996(8.9)	773(8.1)	223(13.1)	
≥ 28	189(1.7)	95(1)	94(5.5)	*< 0.001
Mean temperature-°C	37.11 ± 0.74	37.1 ± 0.73	37.2 ± 0.8	*< 0.001
Symptoms number. (%)				
Fever	7785(39.8)	6876(40.4)	909(30.5)	*< 0.001
Cough	8984(45.9)	8044(47.3)	940(36.7)	*< 0.001
Dyspnea	8349(42.6)	6618(38.9)	1731(67.6)	*< 0.001
Chest pain	943(5.1)	840(5.3)	103(4.1)	0.012*
Stomach ache	687(3.7)	684(4)	39(1.6)	< 0.001
Nausea	352(1.8)	326(1.9)	26(1)	*0.001
Vomiting	1007(5.4)	894(5.6)	113(4.5)	*0.026
Diarrhea	682(3.7)	630(3.9)	52(2.1)	*< 0.001
Loss of appetite	2042(11)	1731(10.8)	311(12.4)	*0.019
Muscular. Pain	5865(30)	5296(31.1)	569(22.2)	*< 0.001
Paresis	132(0.7)	93(0.6)	39(1.6)	*< 0.001
Plegia	59(0.3)	46(0.3)	13(0.5)	0.06
Vertigo	500(2.7)	459(2.9)	41(1.6)	*< 0.001
Seizure	64(0.3)	42(0.2)	23(0.9)	*< 0.001
Insomnia	352(1.8)	326(1.9)	26(1)	*0.001
Unconsciousness	872(4.5)	382(2.2)	490(19.1)	*< 0.001
Headache	1644(9)	1509(9.5)	135(5.4)	*< 0.001
Loss appetite	2042(11)	1731(10.8)	311(12.4)	*0.019
Smell impairment	352(1.8)	326(1.9)	26(1)	*0.001
Taste impairment	198(1)	181(1.1)	17(0.7)	0.059
Skin sign	30(0.2)	27(0.2)	3(0.1)	0.55
GI symptom	3534(19.1)	3112(19.4)	422(16.8)	*0.002
Respiratory symptom	13,950(73.2)	11,870(71.8)	2080(82.1)	*< 0.001
Nervous system	157(0.8)	111(0.7)	46(1.8)	*< 0.001
Comorbidity				
Cardiovascular disease	1450(7.4)	1068(6.3)	382(14.9)	*< 0.001
Hypertension	2236(11.4)	1715(10.)	521(20.4)	*< 0.001
Diabetes mellitus	2267(11.6)	1746(10.3)	521(20.4)	*< 0.001
Cancer	218(1.1)	155(0.9)	63(2.5)	*< 0.001
COPD (Chronic Obstructive Pulmonary Disease)	308(1.6)	194(1.1)	114(4.5)	*< 0.001
CKD (chronic kidney disease)	287(1.5)	212(1.2)	75(2.9)	*< 0.001
Asthma	170(0.9)	133(0.8)	37(1.4)	*0.001
Liver disease	35(0.2)	30(0.2)	5(0.2)	0.8
Neurological disorders	157(0.8)	111(0.7)	46(1.8)	*< 0.001
Hematology	55(0.3)	46(0.3)	9(0.4)	0.46
HIV	8(0.0004)	7(0)	1(0)	0.96
Congenital disorder	15(0.1)	13(0.1)	2(0.1)	0.97
Pregnancy	123(0.6)	121(0.7)	2(1.6)	*< 0.001
Smoking	359(1.8)	293(1.4)	66(2.6)	*0.003
Opium	333(1.7)	242(1.4)	91(3.6)	*< 0.001
Early diagnosis based on				
Positive PCR	7828(40)	6575(38.7)	1253(49)	
Abnormal lung CT	6270(32.1)	5427(31.9)	843(33)	
Clinically admission	5464(27.9)	5003(29.4)	461(18)	*< 0.001
Inpatients treatments				
Dialysis (N = 224/393)	155(54)	111(52.4)	44(58.7)	0.34
Chemotherapy	48(38.7)	32(34)	16(53.3)	0.059
Oxygen therapy	3949(31.2)	3110(29.1)	839(42.2)	*< 0.001
Hospital outcome				
Death	1787(9.1)	819(4.8)	968(37.8)	
Recovery	17,789(90.9)	16,198(95.2)	1591(62.2)	*< 0.001

*P < 0.05.

Of the total patients, 13.1% experienced severe COVID-19 infection. Table 2 shows that patients $\ge$ 65 years of age experienced more severe infections with COVID-19 than younger patients (*P* < 0.001). Patients with a history of the COVID-19 infection and those exposed to other patients experienced more severe infections with COVID-19 (*P* < 0.001). Most clinical complaints in severe cases were related to respiratory symptoms, and the lowest was associated with nervous system symptoms. Patients with severe COVID-19 infection reported more clinical signs associated with the respiratory, gastrointestinal, and nervous systems than non-severe cases (*P* < 0.01).

Accordingly, the most common clinical symptoms observed in patients with severe COVID-19 included anesthesia, seizures, paralysis, pelegia, and shortness of breath, while patients with a history of COPD underlying disease, neurological disorders, cancer, drugs, cardiovascular disease, hypertension, diabetes, and asthma were more likely to develop severe COVID-19 (*P* < 0.01).

The mean body temperature of patients with severe COVID-19 was significantly higher than that of non-severe cases of COVID-19 (*P* < 0.001). Patients treated with oxygen therapy had more severe cases of infection than those without oxygen therapy (*P* < 0.001).

Most patients were admitted with a positive PCR diagnostic test based on the hospitalization indication. According to the hospitalization indication, more than 16% of patients admitted with a positive PCR diagnostic test experienced severe COVID-19 infection (*P* < 0.001). The prevalence of death was significantly higher in severe (37.8%) compared to non-severe patients (4.8%) (*P* < 0.001, Fig. 1). More women were hospitalized with a positive PCR test and men with a positive CT scan (*P* < 0.001).Table 3 show means and medians for survival time of hospitalization from admission to death/discharge in hospitalized patients' death.

**Table 3:** Means and medians for survival time.

Variables	Mean		Median		*P*-value	Cox Model	
	Estimate ± SE	95% CI	Estimate ± SE	95% C		HR 95% CI	*P*-value
Age							
< 65	41.84 ± 2.53	36.88–46.8	30 ± 1.95	26.18–33.83		Ref	
≥ 65	21.85 ± 1.075	19.71–23.96	16 ± 0.42	15.18–16.82	*< 0.001	2.1(1.93–2.72)	*< 0.001
Sex							
Male	31.2 ± 1.79	27.68–34.72	21 ± 0.86	19.31–22.68		Ref	
Female	30.78 ± 1.85	27.16–34.41	21 ± 0.85	19.34–22.66	0.14	0.95(0.88–1.03)	0.26
Pandemic Waves							
1th Wave	34.42 ± 2.51	29.49–39.34	23 ± 1.93	19.23–26.77		Ref	
2th Wave	37.79 ± 4.33	29.30–46.28	28 ± 1.77	24.53–31.47		1.15(0.97–1.36)	0.1
3th Wave	26.86 ± 1.47	23.98–29.73	19 ± 0.5	18.02–19.98		1.65(1.46–1.87)	*< 0.001
4th Wave	13.22 ± 0.54	12.15–14.28	15 ± 1.94	11.19–18.81	*< 0.001	2.14 (1.7–2.71)	*< 0.001
Severity							
Yes	18.83 ± 1.02	16.84–20.83	13 ± 0.45	12.13–13.87		Ref	
No	36.27 ± 2.55	31.27–41.26	25 ± 1.69	21.69–28.31	*< 0.001	4.01(3.65–4.42)	*< 0.001
Overall	30.86 ± 1.29	28.33–33.4	21 ± 0	19.82–22.18			

*P < 0.001, Log-rank test.

Table 3 presents the median length of hospitalization and the risk of severe COVID-19 infection. According to the results of the Kaplan–Meier method, the median length of hospitalization from the time of admission to discharge or death in the hospital (IQR: 13–41) was estimated to be 21 days. As shown by the results of the log-rank test, the median hospital survival was significantly lower in patients aged $\ge 65$ years (16 days) compared to younger patients (30 days) and in severe cases of COVID-19 (13 days) compared to non-severe cases (25 days) (*P* < 0.001). Also, the median hospital survival was significantly different between the pandemic waves, decreasing by 19 and 15 days in the third and early fourth waves, respectively (*P* < 0.001). The results of univariate Cox regression showed that the risk of contracting severe COVID-19 in hospitalized patients significantly increased by 1.65 and 2.14 times in the third and early fourth waves. Also, the risk of contracting severe COVID-19 was significantly higher in patients aged $\ge$ 65 years (HR = 2.1) than in younger people (*P* < 0.001).

**Figure 2:**
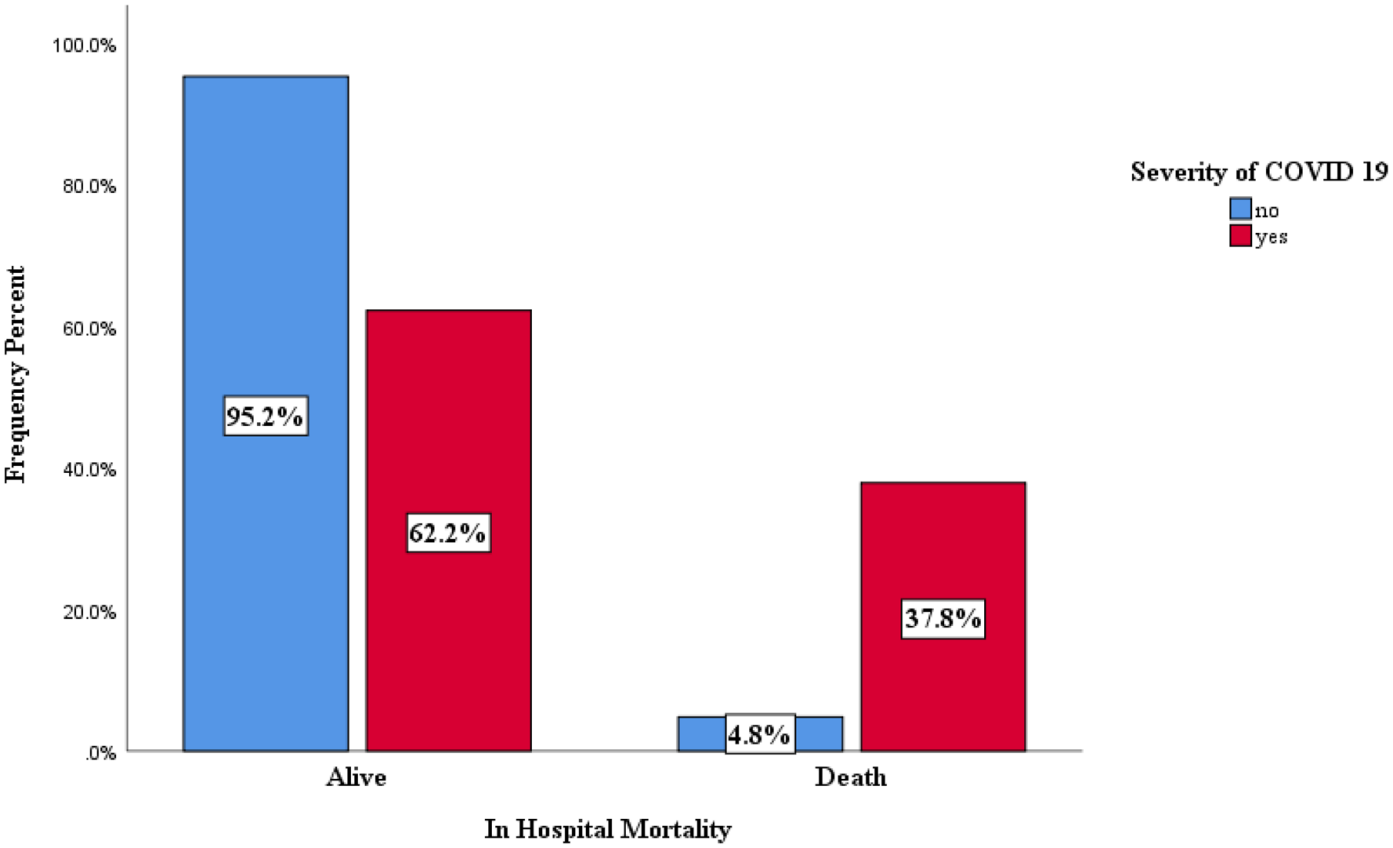
Shows frequency distribution of in-hospital mortality of COVID-19 based to severity disease.

**Figure 3:**
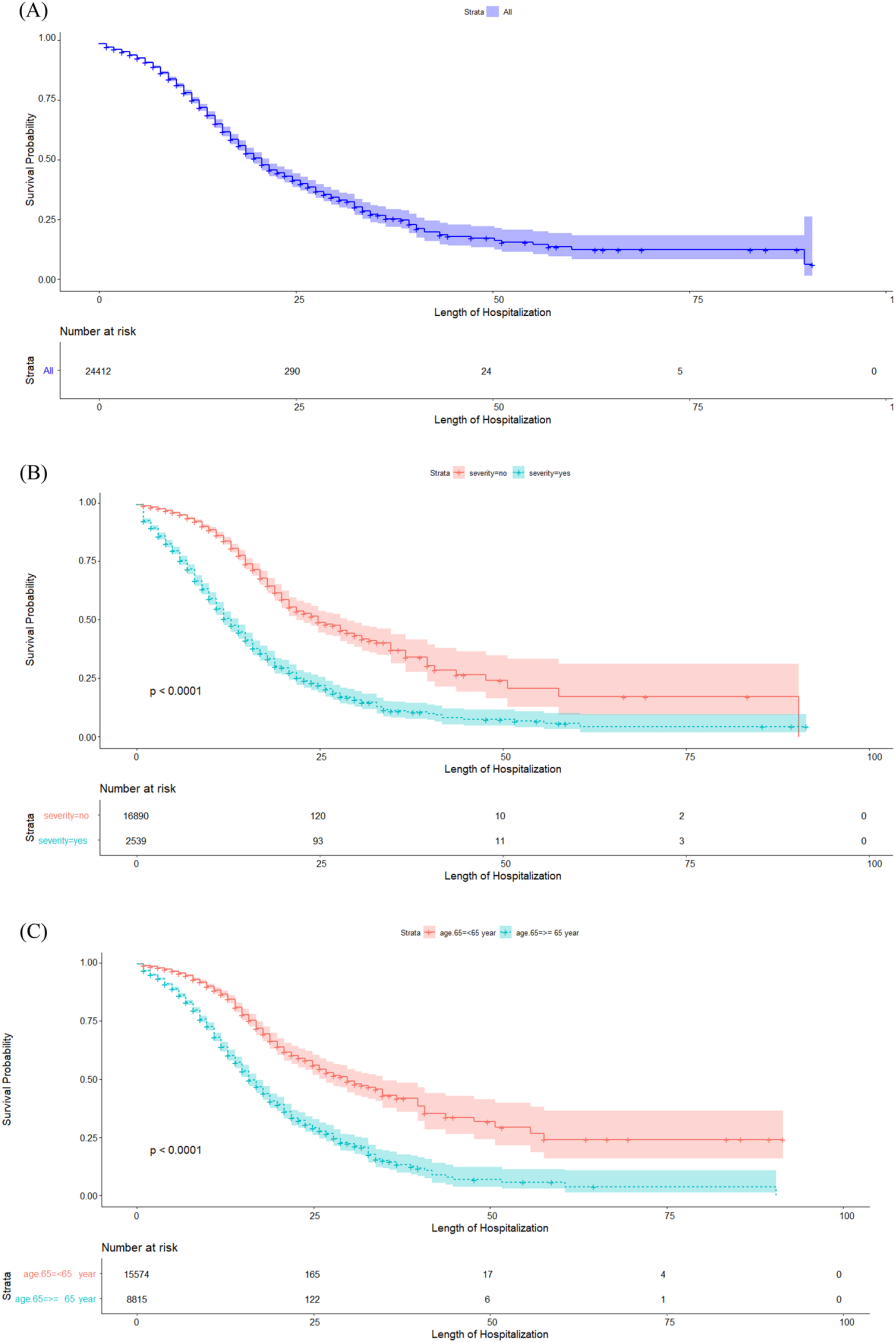

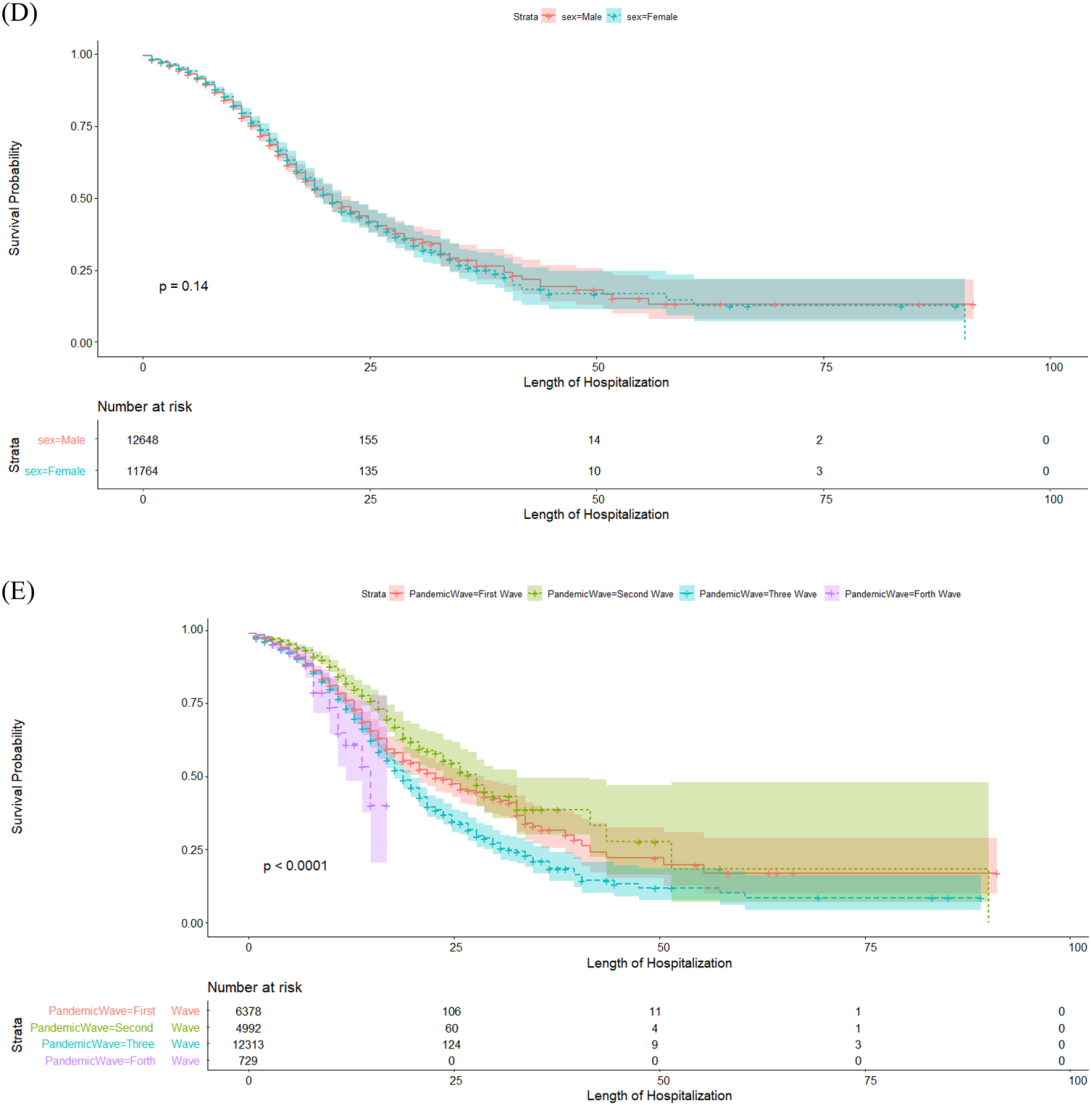
The median length of hospitalization from admission to death/discharge in hospitalized patients' death(Graph A) using the Kaplan-Meir method based on severity(Graph B), age(Graph C), sex(Graph D), pandemic waves(Graph E).

Figure 2 presents the median length of hospitalization from admission to death/discharge in hospitalized patients' death using the Kaplan-Meir method based on age, sex, pandemic waves, severity (Fig. 3).

## Discussion

The data set used in this study was collected from the date of onset of the COVID-19 pandemic from February 22, 2020, to 13 February 13, 2021 from the MCMC hospital registration system. During this period, Iran has identified three waves of the COVID-19 pandemic with the predominant strain of the Wuhan virus, along with the fourth COVID-19 wave in late February 2021. This study described the clinical features and hospital survival of patients based on age, gender, and severity of COVID-19 during the 12 months following the onset of the pandemic.

Our analyses showed that out of 24,563,000 patients with COVID-19, admitted until February 13, 2021, 2185 patients (8.9%) died, and the median survival of the hospital was estimated to be 21 days using the Kaplan-Mayer method. Also, 2559 patients (13.1%) were identified as severe cases of COVID-19. The majority of patients were admitted with a positive PCR test. As observed, the gender distribution of COVID-19 was similar to other studies, with men were more affected than women^8,19^. According to the systematic review and meta-analysis report on 15,828 cases of COVID-19, the prevalence of severe and critical cases was 17.84% and 4.9%, respectively^9^. However, our study showed lower values (13.1%), which may be due to the lack of data related to the hospitalization in the intensive care unit. The severity and hospital mortality of COVID-19 were also affected by gender ^20^. In line with other studies, men experienced a worse prognosis than women^21,22^.

The World Health Report has shown 3.0–8.35 higher mortality rate for men than women^23^. Also, according to evidence from different studies, biological differences in gender affect the severity and outcomes of COVID-19, with men experiencing more severe and worse outcomes ^24–26^. Our data also showed that the mean age men who died was significantly 2 years higher than the age of the deceased women, highlighting the role of age in the severity of COVID-19 outcomes in line with other studies ^22,27^. Also, differences in age, gender, ethnicity, geographical location, and social and cultural structure affect the severity of the disease and its mortality^28,29^.

The most common underlying diseases in hospitalized COVID-19 patients were diabetes, hypertension and heart disease^30,31^. were more at risk of severe COVID-19 cases than others^32^.

Our findings, similar to previous studies, showed that the most common clinical symptoms were fever, cough, and shortness of breath^2,8,33,34^. The prevalence of clinical signs of fever^35^, anesthesia, and the headache was significantly higher in men, while women reported higher prevalence of chest pain, nausea, vomiting, muscle pain, plegia, dizziness, and skin symptoms. In general, respiratory, gastrointestinal, and neurological symptoms were more common in men than women, but the prevalence of clinical symptoms was not affected by gender. Wang et al. reported that clinical signs of fever, cough, and chills were more common in men. Only 35% of patients reported a history of contact with COVID-19 patients, with men showing a significantly higher likelihood for such a contact than women. Besides, these patients experienced severe cases COVID-19 about three time more than the others ^36^. Another study in the early stages of the epidemic in China reported that 23% of severe cases had a history of exposure to areas of epidemic onset^20^. Another preliminary study from China also reported that 72.3% of those infected were in contact with Wuhan residents^37^. In another study, Hong et al. found that 70% of patients had a history of close contact with COVID-19 patients^36^. The results showed that people who had close contact with the patients two days before and three days after the onset of the symptoms were 3.1 times more likely to be at high risk^38^. Compared with asymptomatic patients, those exposed to patients with mild symptoms were 4 and 3.4 times more likely to be at risk than those exposed to moderate cases of the disease^38^. The results showed that the patient's viral load decreased to its full two days before the onset of symptoms and after one week^38^.

. Previous history of the COVID-19 infection was correlated with severe COVID-19 disease. Studies have shown that the cause of the COVID-19 re-infection has been the emergence of new genetic strains, with CDC (2020) reporting a 45-day interval between re-infections^39^ and Tang et al. reporting 19 days^40^.

Duration of hospitalization has been reported based on the average or median in different studies. In a study by Nirmala while, the average length of hospital stay was between 4 to 53 days in 45 hospitals in China and 4–21 in studies outside China, as found in a meta-analytical study with 52 studies (46 studies from China)^43^ Also, the median length of hospital stay was 7 days (in a study from Iran^44^, 12 days in a study by Nirmala ^45^, and 12.4 days using the AF methods^46^.

We found that the length of hospitalization of COVID-19 patients was influenced by gender. Consistent with other studies, our results showed that the duration of hospitalization was longer in severe than non-severe cases^27^, with patients who experienced severe cases of COVID-19 hospitalized three days more than those without severe COVID-19. The length of hospitalization has been reported differently in various studies, and factors such as the severity of the disease, the time from the onset to the diagnosis, age over 45 years, residential area^47^, previous underlying medical conditions, and the location of the infection^48^ affect the length of hospital stay of COVID-19 patients.

Our findings showed that most of patients had a respiratory rate of 14–18 beats per minute, . CT scans also showed about 91% of patients with lung abnormalities Our findings showed that the results of positive CT scans of patients with severe cases of COVID-19 lung involvement were significantly higher than the others.. One of the the essential tools for assessing of COVID-19 severity is the CT scan results of the lungs^27^. Studies have also shown that the respiratory pattern in COVID-19 is not similar to that of the flu and cold, according to which people with COVID-19 have faster breathing due to shortness of breath^8,38^.

Liu et al. noted that patients with severe COVID-19 infections were more prone to faster respiration^8^ and higher respiration rates. According to another study, a decrease in the vital respiratory capacity was observed in 65.4%, while 18.8% of patients represented an abnormal respiratory pattern^38^.

Previous studies have reported that decreased blood oxygen levels were associated with increased severity of COVID-19^8,41^. Studies have shown that the objective symptoms of respiratory distress—oxygen saturation and respiratory rate—are associated with worse outcomes, such as a significant increase in mortality^27^. Chatterjee et al. reported that the chance of mortality in patients with a respiratory rhythm of > 22 beats per minute was 2.3–9.1 times higher than in patients with a normal respiratory rhythm (< 20 beats per minute)^27^. The results of these studies confirm our epidemiological findings.

The mean body temperature of men compared with women and the body temperature of patients with severe COVID-19 also increased significantly compared to the others.. One of the most common clinical signs of COVID-19 infection is fever, which leads to an increase in body temperature^49,50^ and can be considered one of the screening tools to diagnose COVID-19 infection in communities^50^. Wang et al. reported that severe cases of COVID-19 increased significantly in patients with febrile symptoms^50^, while another study reported that severe cases of COVID-19 had higher body temperature^8^. In another study, it was reported that the mortality rate in patients with an average body temperature of more > than 40° C was 42% ^51^ which confirms our findings.

About 5% of the COVID-19 patients admitted to the hospital received treatment with mechanical ventilation.. In other studies, age was high in patients undergoing mechanical ventilation, and elderly patients suffered from underlying diseases, etc.^52^. Our data showed that patients treated with mechanical ventilation were, average nine years older than those without mechanical ventilation treatment.Patients treated with mechanical ventilation experience higher mortality^53^. Consistent with our study, mortality was higher in patients with severe COVID-19. Men also experienced more cases of COVID-19 than women on dialysis.

The results of our study showed that most cases occurred in the third wave of COVID-19 (50.1%) with 12,318 infections, according to which the distribution of severe cases of COVID-19 increased significantly from 7.6% in the first wave to 9.4% during the second wave. It then decreased during the third wave (15.7%) and then decreased significantly at the beginning of the fourth wave 13% in late February. As seen in each wave, the prevalence severs of COVID-19 was inversely related to number of admitted patients.

However, the gender distribution did not differ significantly during the COVID-19 pandemic waves. In addition to mutations in the COVID-19 virus and changes in its transmission, other factors such as social events, the end of quarantine, gathering in enclosed places, etc. led to numerous waves of COVID-19 in many countries with different intensities and characteristics^1^. As spainish study reported that the number of hospitalizations increased from 204 in the first wave to 264 in the second wave^1^. On the other hand, the duration of hospitalization in the second wave was shorter than in the first wave^1^.

Based on the results of the log-rank test, the duration of hospitalization decreased significantly from 25–28 days in the first and second waves to 19 days in the third and 15 days in the early fourth wave in late February. It seems, it was because of admission indications were changed during first year of COVID-19 pandemic so that in the later wave the sever patients were admitted more than in the early wave. This changes in severity of COVID-19 can be due to admission criteria changes during pandemic.

Our analysis showed that the risk of severe cases was 1.65 and 2.14 times higher than the first wave of COVID-19 in the third and early fourth wave to the end of February 2021 can be explained by admission criteria strategies changes during pandemia and previous exposure to COVID-19 as predictors of severity of disease. Also, the risk of severe cases of COVID-19 was 2.1 times higher in patients aged $\ge 65$ years than in younger people.

## Conclusion

According to evidence obtained in our study, the mortality rate and severity of COVID-19 were comparative with world experiences. The incidence and mortality rate was higher in the elderly ,men, those with a history of underlying diseases. Our data also showed that patients with a previous history of COVID-19 had a more severe experience of COVID-19. In addition, based on the hospitalization indication, the hospitalized patients with positive PCR test experienced the most severe COVID-19 . During first year of pandemia we saw the risk of severe cases in the third and early fourth wave were 1.654 and 2.148 times higher than the first wave of COVID-19.

### Strengths and weaknesses

One of the limitations of the present study is the lack of data related to hospitalization in the intensive care unit, which is one of the cases in the category of diagnosis of severe cases of COVID-19 and may have led to underestimating the prevalence of severe cases of COVID-19 in our study. Other topics include failure to record the time of the onset of clinical symptoms as one of the factors affecting the severity of COVID-19 is hospitalization. One of the strengths of the present study is the selected duration of time and the high volume of data.

## Data Availability

Availability of these data, was under license for the current study, and so is not publicly available. The data that support the findings of this study are available from Shahid Sadoughi University of Medical Sciences but restrictions apply to the availability of these data, which were used under license for the current study, and so are not publicly available. Data are however available from the authors upon reasonable request and with permission of Shahid Sadoughi University of Medical Sciences. For more information referred to Dr Namayandeh by Email: drnamayandeh@gmail.com.
